# High-Intensity 1064 nm Laser Therapy for Temporomandibular Joint Pain: A Prospective Multicenter Study of TriHIL™ Technology

**DOI:** 10.7759/cureus.116033

**Published:** 2026-09-10

**Authors:** Tina Chandra, Shireen Dhanani, Jim W McCreight

**Affiliations:** 1 Dentistry, Chandra Wellness Center, Ocala, USA; 2 Dentistry, The Dental Touch, Leesburg, USA; 3 Dentistry, McCreight Progressive Dentistry, Steamboat Springs, USA

**Keywords:** 1064 nm laser, high-intensity laser therapy, laser therapy, mandibular range of motion, noninvasive treatment, orofacial pain, temporomandibular disorders, temporomandibular joint pain, tmd disability index

## Abstract

Background and objective

Temporomandibular disorders (TMDs) are common and can substantially impair quality of life. Conventional treatments are often limited by adverse effects, poor compliance, or lengthy treatment courses. A high-intensity 1064 nm laser system with TriHIL™ technology (BTL Industries, Boston, MA, USA) may offer a noninvasive alternative with a shorter treatment course. The objective of this study was to evaluate the safety and efficacy of a high-intensity 1064 nm laser system with TriHIL technology in alleviating temporomandibular joint (TMJ) pain and dysfunction.

Methods

This prospective, multicenter, single-arm study enrolled 31 adults (28 women and three men; mean age 50.2 ± 14.5 years) with TMJ pain across three clinical sites. Subjects received four 20-minute treatment sessions spaced two to 10 days apart. Pain (Visual Analog Scale, VAS), mandibular range of motion (ROM), and TMD Disability Index (TMD-DI) were assessed at baseline, posttreatment, and at one-month and three-month follow-up (1M FU and 3M FU). Treatment tolerability (Therapy Comfort Questionnaire, TCQ) was assessed posttreatment, and patient satisfaction (Subject Satisfaction Questionnaire, SSQ) was assessed at posttreatment and at 1M FU and 3M FU.

Results

All 31 subjects completed the four-session protocol and all follow-up visits. At 3M FU, mean spontaneous VAS pain decreased from 4.37 ± 2.25 to 1.52 ± 2.11 (a relative reduction of 65.3%, p < 0.001), and VAS during palpation decreased from 5.92 ± 2.35 to 1.87 ± 2.14 (a relative reduction of 68.4%, p < 0.001). TMD-DI scores decreased from 20.6 ± 11.6% to 11.3 ± 13.2% (a relative reduction of 45.3%, p < 0.001). Lateral mandibular ROM improved significantly at all time points (n = 24, +3.2 mm at 3M FU, p = 0.044). Overall, 24 (77.4%) subjects remained satisfied with treatment outcomes at 3M FU. On the TCQ, 30 (96.8%) subjects rated the treatment as comfortable, with a mean intraprocedural pain score of 0.13/10. No device-related adverse events occurred.

Conclusions

A four-session protocol with the TriHIL technology was associated with substantial and durable reductions in TMJ pain and disability with no device-related adverse events, supporting its use as a noninvasive option for the management of TMJ pain.

## Introduction

The temporomandibular joint (TMJ) enables the multidimensional jaw movements involved in mastication and speech and ranks among the most anatomically complex and frequently loaded joints in the human body [1]. This combination of intricate anatomy and continuous mechanical demand makes the TMJ highly susceptible to dysfunction. Temporomandibular disorders (TMDs) affect approximately 29.5% of the global population [2] and manifest as jaw pain, restricted chewing and speech, joint clicking, and headaches. Beyond these symptoms, TMDs substantially impair quality of life, disrupting eating, communication, and sleep, and frequently progress into a chronic condition that requires sustained clinical management [3].

The etiology of TMDs is multifactorial, encompassing mechanical stress, parafunctional habits (e.g., bruxism), trauma, and psychological factors [4]. Initial management typically relies on conservative approaches, including nonsteroidal anti-inflammatory drugs (NSAIDs), occlusal splints, and physical therapy, while refractory cases may require more invasive interventions such as arthrocentesis, intra-articular injections, or surgery [5]. Increasingly, energy-based modalities have emerged as noninvasive alternatives, namely, low-level laser therapy (LLLT), transcutaneous electrical nerve stimulation (TENS), and therapeutic ultrasound [6].

Each of these approaches, however, carries notable limitations. Pharmacotherapy offers only symptomatic relief and is associated with adverse effects and dependency risk [7]; occlusal splints suffer from poor compliance and fail to address underlying inflammation, and surgical interventions remain invasive and reserved as a last resort [8]. Among emerging modalities (TENS, therapeutic ultrasound, and LLLT), durable long-term outcomes have been inconsistently demonstrated, with most evidence supporting only short-term symptomatic relief. LLLT in particular is constrained by shallow tissue penetration, prolonged treatment courses, and high operator dependency [6,9]. Although high-intensity laser therapy has shown promise in addressing these constraints, existing protocols vary widely in wavelength, dose, and delivery method, and durability beyond the immediate posttreatment period remains insufficiently characterized [10,11]. These shortcomings highlight the need for a noninvasive, deep-penetrating, reproducible, and time-efficient treatment that delivers durable outcomes in TMD-related pain.

A high-intensity laser system with TriHIL™ technology (BTL Industries, Boston, MA, USA), delivering up to 30 W of average power at 1064 nm, may address these limitations. Among therapeutic laser wavelengths, 1064 nm provides one of the deepest tissue penetration [10,12,13], reaching the TMJ capsule, articular disc, and surrounding masticatory muscles. Its therapeutic effects are proposed to arise from multiple complementary mechanisms: photomechanical analgesia consistent with the gate control theory [14], photobiomodulation enhancing adenosine triphosphate production and tissue repair [15], anti-inflammatory action through cytokine modulation [15], and thermal-mediated vasodilation and deep tissue relaxation [16].

The present study evaluated the safety and efficacy of a novel TriHIL technology in the treatment of TMJ pain and dysfunction. To our knowledge, this is the first prospective multicenter study to evaluate a high-power 1064 nm laser with TriHIL™ technology for TMJ pain and among the few to report the durability of multidomain outcomes (pain, disability, range of motion (ROM), and satisfaction) at three-month follow-up, addressing the limited durability data that characterize existing high-intensity laser protocols.

## Materials and methods

Study design and ethics

This prospective, single-arm, multicenter study was conducted at three clinical sites between August 2024 and May 2025, in accordance with the Declaration of Helsinki. The study protocol was approved by the Advarra Institutional Review Board (approval Pro00080615), and all participants provided written informed consent prior to enrollment. The trial was registered at ClinicalTrials.gov (NCT07624539).

Participant selection

Adults aged ≥ 22 years presenting with TMJ pain were recruited. To be included, candidates had to commit to all scheduled study visits, maintain their established diet, exercise, and pain management regimens, and agree to abstain from initiating any new TMJ treatments for the duration of the study; additionally, those of childbearing potential were required to use active birth control.

Individuals were excluded if they were pregnant, presented with severe systemic diseases, or had a history of bleeding disorders or anticoagulant therapy. Further exclusion criteria included photosensitivity, local tissue abnormalities in the treatment area (tattoos, hyperpigmentation, hemorrhage, or sensory loss), and any active or past malignancy. Finally, subjects were disqualified if they had received radiotherapy within the previous six months or had undergone local TMJ injections or corticosteroid treatments within the previous three months.

Treatment protocol

Preparation for treatment included shaving facial hair and cleaning the treatment area with a gentle cleanser. Treatment was initiated only after complete drying of the area. All subjects received four treatments with the EMVITAL^®^ device (BTL Industries) employing the TriHIL technology, targeted over the TMJ area, lasting 20 minutes and spaced two to 10 days apart. To optimize safety and tolerability, the output intensity was adjusted according to real-time patient feedback. Treatment was delivered using a fixed manufacturer preset (EMVITAL^®^, TriHIL technology; 1064 nm; up to 30 W average power). The detailed dosimetry parameters of this preset are proprietary to the manufacturer and were not disclosed.

Data collection and evaluation

Mandibular ROM, pain levels, and the impact of the disorder on daily life were evaluated at baseline, immediately following the final treatment (post-Tx), and at one-month and three-month follow-up (1M FU and 3M FU). ROM was measured with a disposable Range of Motion Scale (product no. 255-009, Great Lakes Dental Technologies, Towanda, NY, USA) to record three specific metrics: comfortable opening, maximum active opening, and lateral movement. Pain intensity was assessed using a Visual Analog Scale (VAS) [17], ranging from 0 (no pain) to 10 (worst possible pain), for both spontaneous pain and pain elicited by palpation. The impact on daily life was assessed using the TMD Disability Index (TMD-DI) [18], a standardized 10-item self-reported questionnaire; total raw scores (range 0-40, each item scored from 0 to 4) were converted to a percentage of the maximum possible score (0-100%), with higher percentages indicating greater disability. Individual item scores are reported in raw points. Following the final session, treatment tolerability was measured using the study-specific Therapy Comfort Questionnaire (TCQ), which includes a 5-point Likert scale and a Numerical Analog Pain Scale (range 0-10), where 0 denotes no pain, and 10 indicates the worst possible pain (Appendix A). Subject satisfaction and quality of life were assessed via a study-specific 12-item Subject Satisfaction Questionnaire (SSQ) using a 5-point Likert scale (1 = strongly disagree, 5 = strongly agree) at post-Tx, 1M FU, and 3M FU (Appendix B).

Statistical analysis

The primary outcome was TMJ pain (VAS, spontaneous and on palpation); secondary outcomes were mandibular ROM, TMD-DI, patient satisfaction (SSQ), and treatment tolerability and safety (TCQ). As a pilot investigation, the sample size was not determined using a formal power calculation; all eligible participants enrolled across the three sites during the study period were included. Statistical analyses were performed in JASP (Version 0.96.0; JASP Team, University of Amsterdam, Amsterdam, Netherlands). Continuous outcomes were summarized as mean ± standard deviation. Given the ordinal nature of the outcome scales and the sample size, nonparametric tests were used. Within-subject changes across the four time points (baseline, post-Tx, 1M FU, and 3M FU) were assessed using Friedman’s test. When the omnibus Friedman test indicated a significant effect of time, pairwise post hoc comparisons were performed using Conover’s test with Holm correction for multiple comparisons. Spearman’s rank correlation was used to assess associations between baseline-to-3M FU changes in primary outcomes. Tolerability and subgroup comparisons (by pain duration and baseline severity) were assessed descriptively only, given the limited sample sizes within subgroups. A two-sided p < 0.05 was considered statistically significant.

## Results

Subjects

A total of 31 subjects (28 women and three men; mean age 50.2 ± 14.5 years; mean BMI 31.3 ± 6.6; Fitzpatrick skin types I-V [19]) were enrolled across three clinical sites. All subjects completed the four treatment sessions and all follow-up visits. Subjects presented with either chronic (n = 26) or acute (n = 5) TMJ pain.

Pain outcomes

Treatment resulted in significant reductions in TMJ pain at all time points (Figure 1, Table 1). Mean VAS spontaneous pain decreased from 4.37 ± 2.25 to 1.52 ± 2.11 at 3M FU (a relative reduction of 65.3%, p < 0.001), and VAS during palpation decreased from 5.92 ± 2.35 to 1.87 ± 2.14 (a relative reduction of 68.4%, p < 0.001). At 3M FU, 28 (90.3%) subjects reported reduced VAS spontaneous pain and 30 (96.8%) reported reduced pain during palpation (Figure 2).

**Figure 1:**
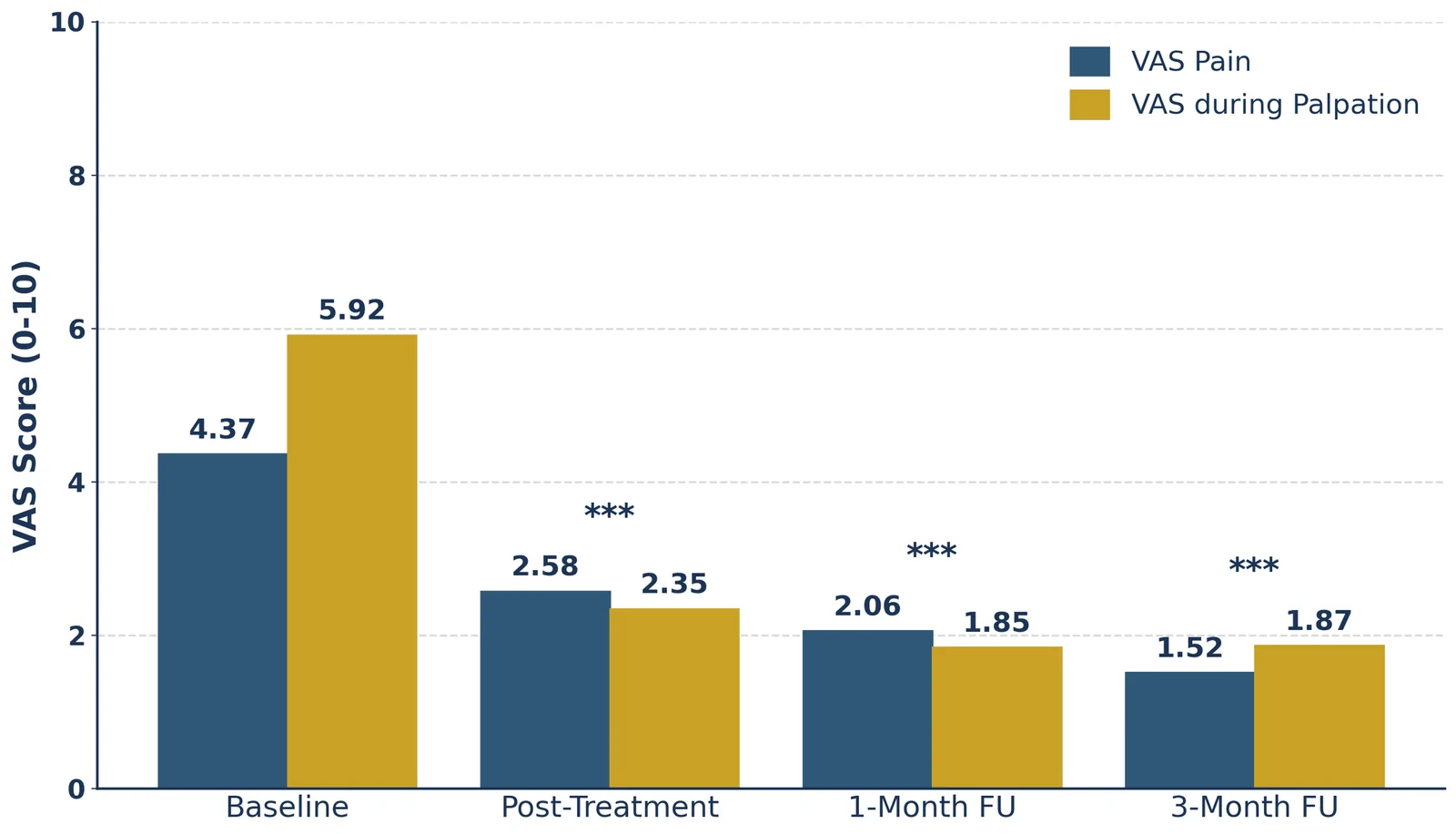
VAS scores for spontaneous pain and pain during TMJ palpation at baseline, posttreatment, and 1M FU and 3M FU. Bars show mean values; n = 31. Asterisks indicate p < 0.001 versus baseline for both measures (Conover’s post hoc test with Holm correction). 1M FU, one-month follow-up; 3M FU, three-month follow-up; FU, follow-up; TMJ, temporomandibular joint; VAS, Visual Analog Scale

**Table 1:** Mean pain, disability, and ROM scores at baseline and follow-up (mean ± SD). Values are mean ± SD; n = 31 unless indicated. * n = 24 (bilateral lateral excursion; missing baseline measurements in seven subjects) 1M FU, one-month follow-up; 3M FU, three-month follow-up; p, omnibus Friedman test across the four time points; post-Tx, immediately after the final treatment session; ROM, range of motion; TMD-DI, Temporomandibular Disorder Disability Index; VAS, Visual Analog Scale

Outcome	Baseline	Post-Tx	1M FU	3M FU	p
VAS spontaneous pain (0-10)	4.37 ± 2.25	2.58 ± 2.42	2.06 ± 1.89	1.52 ± 2.11	<0.001
VAS pain during palpation (0-10)	5.92 ± 2.35	2.35 ± 1.96	1.85 ± 1.80	1.87 ± 2.14	<0.001
TMD-DI disability score (%)	20.6 ± 11.6	12.3 ± 11.0	12.3 ± 14.3	11.3 ± 13.2	<0.001
Comfortable opening (mm)	35.6 ± 7.3	37.9 ± 7.0	36.5 ± 8.0	36.6 ± 8.3	0.041
Maximum active opening (mm)	44.9 ± 6.1	46.8 ± 6.8	46.9 ± 6.2	46.2 ± 7.2	0.266
Lateral ROM (mm)*	17.8 ± 6.0	21.6 ± 5.7	22.7 ± 5.5	21.0 ± 6.0	0.003

**Figure 2:**
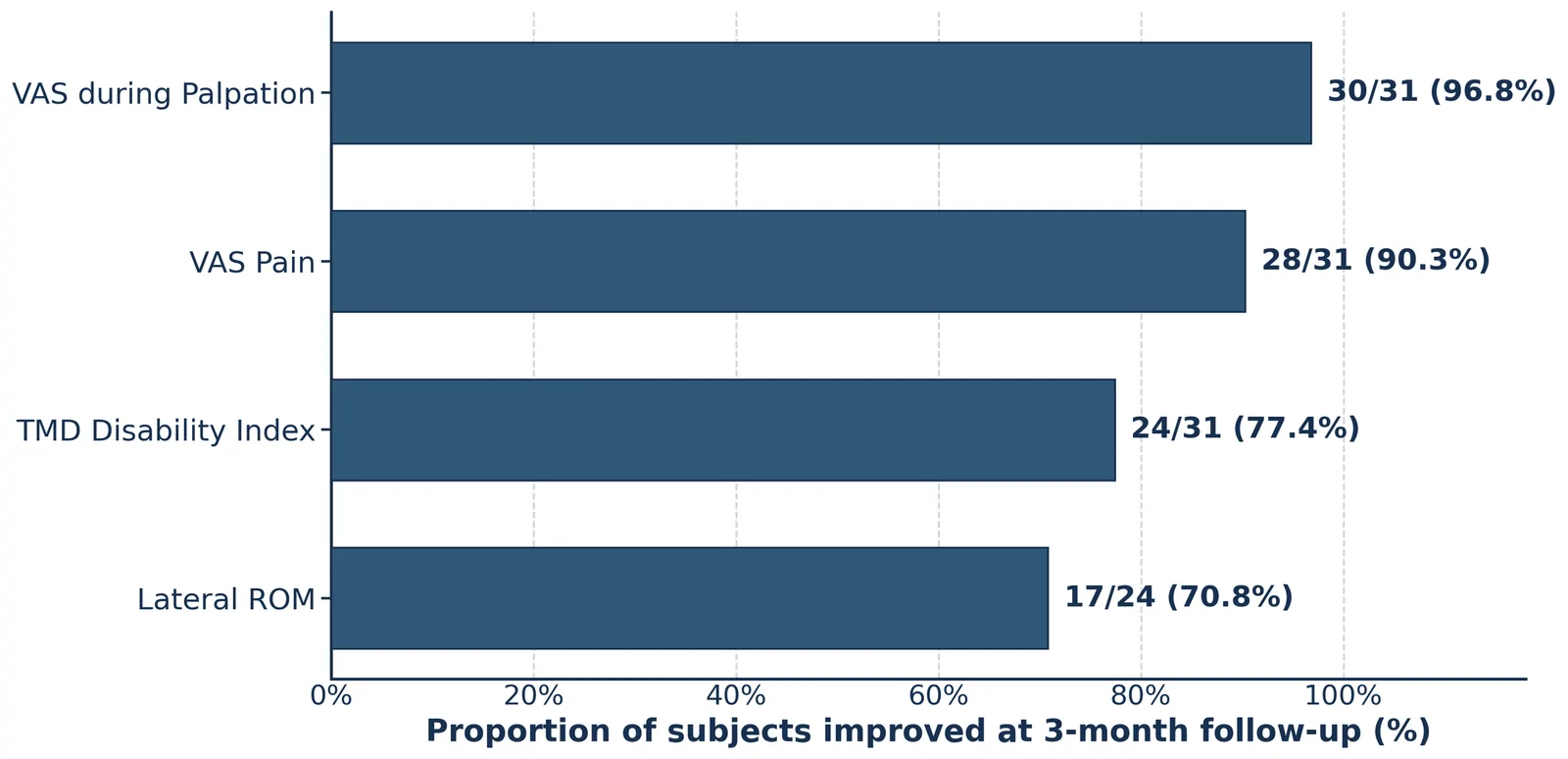
Proportions of subjects with any improvement from baseline at 3M FU across pain, disability, and functional outcomes. Bars show the number and percentage of subjects with any improvement from baseline. Data were available for 31 subjects for all outcomes except lateral ROM (n = 24 due to missing baseline measurements). Outcomes are ordered by magnitude of improvement. 3M FU, three-month follow-up; ROM, range of motion; TMD, temporomandibular disorder; VAS, Visual Analog Scale

At three-month follow-up, a ≥ 2-point reduction was observed in 23 (74.2%) subjects on spontaneous VAS pain and in 26 (83.9%) subjects on VAS during palpation. At baseline, seven subjects reported severe spontaneous TMJ pain (VAS ≥ 7). At 3M FU, six of these seven subjects shifted to a lower pain category (VAS <7), and all seven recorded a decrease in absolute pain scores. For pain during TMJ palpation, 14 subjects presented with severe baseline pain. At 3M FU, 13 of these 14 subjects shifted to a lower pain category, and all 14 recorded a decrease in absolute scores.

A descriptive subgroup analysis based on pain duration (acute, n = 5; chronic, n = 26) suggested differences in both baseline severity and response trajectories. At baseline, the acute subgroup reported higher mean VAS scores than the chronic subgroup for spontaneous pain (4.80 vs. 4.29) and particularly for pain during palpation (7.20 vs. 5.67). At post-Tx, the chronic subgroup showed a greater mean decrease in spontaneous pain VAS (from 4.29 to 2.35) compared with the acute subgroup (from 4.80 to 3.80). By 3M FU, mean pain scores were 1.20 in the acute subgroup and 1.58 in the chronic subgroup. Regarding VAS pain during palpation, the acute subgroup demonstrated a larger mean decrease (from 7.20 to 2.20) at post-Tx compared with the chronic subgroup (from 5.67 to 2.38). At 3M FU, the mean VAS score during palpation was 2.20 in the acute subgroup and 1.81 in the chronic subgroup.

VAS spontaneous pain and VAS during palpation changes were significantly correlated (ρ = 0.644, p < 0.001). Improvements in spontaneous pain at 3M FU were significantly correlated with improvements in TMD-DI (ρ = 0.385, p = 0.032).

Functional outcomes 

The TMD-DI demonstrated significant improvement at all post-baseline time points (all p < 0.001), with mean total scores decreasing from 20.6 ± 11.6% at baseline to 12.3 ± 11.0% posttreatment (a relative reduction of 40.6%), 12.3 ± 14.3% at 1M FU (40.2%), and 11.3 ± 13.2% at 3M FU (45.3%) (Table 1). The proportion of subjects with any improvement from baseline was 24 (77.4%) at 3M FU (Figure 2). At the domain level, the largest mean reductions at 3M FU were observed in mouth opening and yawning (from 1.58 to 0.77 points) and coping without medication (from 1.39 to 0.61 points), followed by sexual function (from 0.65 to 0.29 points) and communication (from 0.61 to 0.29 points) (Figure 3). Furthermore, the proportion of subjects with a TMD-DI score in the lowest disability band (≤ 20% of the maximum score) increased from 18 (58.1%) at baseline to 26 (83.9%) at 3M FU.

**Figure 3:**
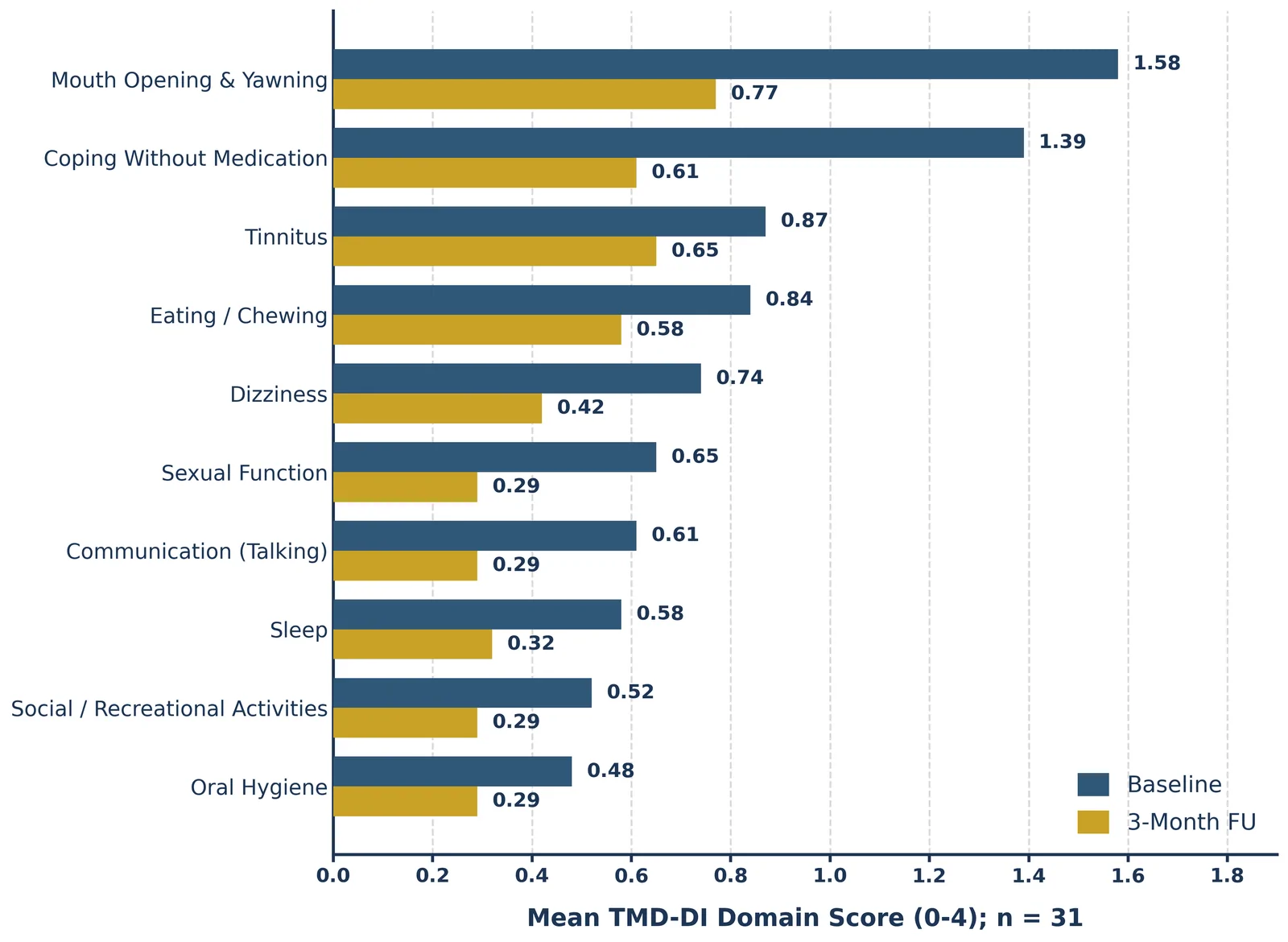
Mean TMD-DI domain scores at baseline and 3M FU. Bars show mean domain scores at baseline and at 3M FU (n = 31 for all domains). Each item is scored from 0 to 4, with higher scores indicating greater disability. Domains are ordered by baseline score. 3M FU, three-month follow-up; FU, follow-up; TMD-DI, Temporomandibular Disorder Disability Index

Lateral ROM (n = 24) increased significantly at all time points, with mean gains of +3.9 mm at post-Tx (p < 0.001) and +3.2 mm at 3M FU (p = 0.044) (Table 1). At baseline, seven of 24 subjects (29.2%) presented with impaired lateral excursion (<7 mm per Helkimo criteria [20]) on at least one side. Any improvement from baseline was observed in 17 (70.8%) subjects at 3M FU (Figure 2). A significant inverse correlation was observed between baseline values and the degree of improvement at 3M FU (Spearman’s ρ = −0.529, p = 0.008).

Mandibular comfortable opening increased significantly at post-Tx (+2.4 mm, p = 0.028), but the effect was not sustained at 3M FU (+1.0 mm, p = 0.672). For maximum active mandibular opening, no time point differed significantly from baseline (post-Tx: +1.8 mm, p = 0.561; 3M FU: +1.2 mm, p = 1.000). At baseline, six of 31 subjects (19.4%) presented with restricted active opening (<40 mm), and six (19.4%) presented with restricted comfortable opening (<30 mm), per Helkimo criteria [20].

Satisfaction, tolerability, and safety

Subject satisfaction was evaluated using the SSQ questionnaire at all posttreatment time points. Immediately after the final treatment session, 26 (83.9%) subjects were satisfied with the overall treatment outcomes, 30 (96.8%) reported that their jaw felt more relaxed, and 25 (80.6%) stated that moving their mouth was easier. At the final 3M FU, 24 (77.4%) subjects remained satisfied with the treatment outcomes, 25 (80.6%) reported a more relaxed jaw, and 19 (61.3%) reported easier mouth movement (Table 2).

**Table 2:** SSQ: agreement per item at post-Tx and 3M FU. Values are the number (percentage) of subjects who agreed (rating ≥ 4 on a 5-point Likert scale) with each statement; n = 31. 3M FU, three-month follow-up; post-Tx, immediately after the final treatment session; SSQ, Subject Satisfaction Questionnaire; TMJ, temporomandibular joint

SSQ item	Post-Tx, n (%)	3M FU, n (%)
Jaw feels more relaxed	30 (96.8%)	25 (80.6%)
Satisfied with treatment outcomes	26 (83.9%)	24 (77.4%)
Less frustrated with TMJ pain	20 (64.5%)	23 (74.2%)
Reduced jaw stiffness	22 (71.0%)	22 (71.0%)
Less TMJ (jaw) pain	22 (71.0%)	21 (67.7%)
Less pain when opening mouth/yawning	19 (61.3%)	20 (64.5%)
Easier mouth movement	25 (80.6%)	19 (61.3%)
Less pain when eating/chewing	22 (71.0%)	19 (61.3%)
Jaw feels less tender	24 (77.4%)	18 (58.1%)
Fewer headaches	12 (38.7%)	16 (51.6%)
Reduced jaw clicking	13 (41.9%)	15 (48.4%)
Less neck pain	18 (58.1%)	13 (41.9%)

Exploratory subgroup analyses of patient satisfaction were conducted based on pain duration and baseline severity. Among subjects with acute TMJ pain (n = 5), all five reported a more relaxed jaw and overall satisfaction with the treatment outcomes at all time points. Among subjects presenting with severe baseline pain (VAS ≥ 7), all seven with severe spontaneous pain and all 14 with severe palpatory pain (100% in each subgroup) reported that their jaw felt more relaxed at post-Tx. At 3M FU, five of seven subjects (71.4%) in the severe spontaneous pain subgroup and 12 of 14 subjects (85.7%) in the severe palpatory pain subgroup remained satisfied with the overall treatment outcomes.

Regarding safety, no serious adverse events were reported during the study. One mild adverse event, consisting of angular cheilitis, was recorded; however, it was determined to be unrelated to the investigational device or the treatment procedure. Overall, 30 subjects (96.8%) rated the treatment as comfortable on the TCQ, with a mean intraprocedural pain score of 0.13/10.

## Discussion

This prospective multicenter study evaluated the safety and efficacy of the TriHIL technology for the treatment of TMJ pain and dysfunction. After four treatment sessions, subjects experienced substantial and durable reductions in pain and TMD-related disability, sustained through the 3M FU. These improvements were paralleled by gains in lateral mandibular ROM and high levels of patient-reported satisfaction. Treatment was well tolerated, with no device-related adverse events and complete retention across the study period.

Pain improved substantially after only four treatment sessions, with VAS pain and VAS pain during palpation reduced by 65.3% and 68.4%, respectively, at 3M FU. The rapid onset of analgesia is consistent with the photomechanical effect of pulsed laser emissions, while the durability of pain relief at 3M FU likely reflects deeper tissue-level photobiomodulation and anti-inflammatory effects enabled by the penetration depth of the 1064 nm wavelength [14,15]. The magnitude of pain reduction is consistent with the 60-70% range reported across LLLT trials in TMD [6], achieved here with markedly fewer sessions than the multiweek courses typical of LLLT protocols, a difference attributable to the substantially higher average power (up to 30 W) and deeper penetration compared with low-level systems. The 2.9-point absolute reduction in spontaneous VAS pain at 3M FU exceeds the 2-point minimum clinically important difference (MCID) established for chronic pain rating scales [21]. Exploratory subgroup analyses indicated consistent response across baseline severity and pain duration. Among subjects with severe baseline pain (VAS ≥ 7), six of seven with severe spontaneous pain and 13 of 14 with severe palpatory pain shifted out of the severe category at 3M FU. In analyses by pain duration, pain reduction was observed in both acute (n = 5) and chronic (n = 26) cases, with mean VAS scores converging to similar low levels at 3M FU despite differing baseline severity. While the small subgroup sizes preclude formal comparison, this pattern suggests that the protocol may be applicable across pain duration and baseline severity.

These results compare favorably with established first-line conservative treatments. A placebo-controlled trial of naproxen 500 mg twice daily reported a 74% VAS pain reduction over six weeks, an analgesic magnitude comparable to that observed here but achieved through daily oral medication, with an increased incidence of gastrointestinal adverse events [22]. Hard stabilization splints, a widely prescribed first-line therapy, also reduce pain in myogenous TMD with moderate-quality evidence, though their efficacy depends on continuous nightly wear and shows attenuation over long-term follow-up [23]. For physiotherapy, a systematic review of 20 randomized clinical trials reported short-term mean pain reductions of 1.47 points on a 0-10 scale with manual therapy, which the authors concluded “may not be clinically important” relative to the 2-point MCID [24]. The pain reduction observed in this study exceeds this threshold and was achieved with a fixed four-session protocol, without the daily medication, sustained appliance wear, or repeated rehabilitation visits these alternatives require, and was sustained for three months beyond the end of the intervention.

Beyond pain, treatment was accompanied by clinically meaningful functional recovery. Given that the 1064 nm wavelength delivered by TriHIL technology induces thermal-mediated vasodilation and relaxation in deep tissues [16], we expected these effects on the masticatory system to translate into measurable gains in mandibular ROM and reductions in TMD-related disability. The TMD-DI decreased by 45.3% at 3M FU, with the largest mean reductions in domains directly tied to daily life: mouth opening and yawning, coping without medication, sexual function, and communication. A retrospective study of multimodal physical therapy in a comparable TMD cohort reported a similar 44% TMD-DI reduction [25], but over a mean of nine sessions rather than four, suggesting higher clinical efficiency of the present protocol. Among the largest domain improvements, the reduction in coping without medication indicates a decrease in treatment dependency, with subjects reporting an increased ability to control discomfort without medications or oral orthotics. This is clinically relevant given the systemic risks of long-term NSAID use and the compliance burden associated with long-term appliance wear.

Maximum active mandibular opening did not change significantly at any time point, consistent with a ceiling effect: only six (19.4%) subjects presented with restricted active opening at baseline (<40 mm per Helkimo criteria [20]), and the group-level mean (44.9 ± 6.1 mm) exceeded this threshold by a wide margin, leaving limited room for further improvement. Comfortable mandibular opening was initially reduced relative to active opening (35.6 ± 7.3 mm at baseline), reflecting pain-limited rather than structural restriction. It improved transiently at post-Tx but did not persist at 1M or 3M FU. In contrast, lateral mandibular ROM increased significantly at all time points. Although mean baseline values for left (8.8 ± 3.5 mm) and right (8.9 ± 2.9 mm) lateral excursions fell within the normal Helkimo range (≥ 7 mm) [20], seven of 24 subjects (29.2%) presented with impaired excursion at baseline. The inverse correlation between baseline values and improvement at 3M FU (ρ = −0.529, p = 0.008) indicates that subjects with the most restricted baseline values showed the greatest gains, although regression to the mean cannot be excluded as a contributing factor. The mouth opening and yawning domain of the TMD-DI showed both the highest baseline burden and the largest mean improvement (Figure 3), despite stable absolute vertical measurements. This suggests that the therapy primarily enhanced the quality and comfort of movement rather than extending the anatomical ROM, possibly by reducing fear-avoidance behavior.

The main strengths of this study are its multicenter prospective design, validated multidomain outcomes, 100% completion rate (31 of 31 subjects), and three-month follow-up. The latter addresses a key gap in the existing laser therapy literature for TMD [6], where durability beyond immediate posttreatment effects remains insufficiently characterized. These strengths are tempered by a single-arm, open-label design without a control group and a small sample (n = 31) with a limited acute subgroup. Because the design was single-arm and open-label, the observed improvements should be interpreted with caution. Expectation (placebo) effects associated with a novel energy-based device, the natural history of TMD, including episodic symptom fluctuation and spontaneous remission, and regression to the mean may all have contributed. Outcomes beyond three months remain uncharacterized, which is particularly relevant for chronic TMD. Sham-controlled trials and trials against active comparators, including pharmacotherapy, occlusal appliances, physiotherapy, and orthodontic treatment, with larger samples, extended follow-up, and stratification by TMD subtype are warranted to confirm and refine these findings.

## Conclusions

A four-session protocol with the TriHIL technology was associated with substantial and durable reductions in TMJ pain and TMD-related disability, sustained through three-month follow-up, with high patient satisfaction and no serious adverse events. The combination of clinical efficacy, brief treatment course, and favorable safety profile supports this approach as a noninvasive option for subjects with TMJ pain, particularly those seeking to reduce reliance on pharmacological or appliance-based therapies. Larger randomized controlled trials with extended follow-up are needed to confirm these findings.

## Appendices

Appendix A 

**Table 3:** Therapy Comfort Questionnaire

Therapy Comfort Questionnaire										
Subject ID:										
Date: ____ / ____ / 20____ (MM / DD / YY)										
Please answer the following questions.										
(1) Please indicate your level of agreement with the following statement: “I found the treatment comfortable.”										
☐ Strongly agree										
☐ Agree										
☐ Neither agree nor disagree										
☐ Disagree										
☐ Strongly disagree										
(2) Was the treatment painful?										
☐ No										
☐ Yes										
If yes, please indicate the level of your pain on the Numerical Analog Scale (0-10) below, where 0 represents “no pain” and 10 represents “worst possible pain.”										
0	1	2	3	4	5	6	7	8	9	10
None	Mild			Moderate			Severe			
Rated pain (0-10):										

Appendix B  

**Table 4:** Subject Satisfaction Questionnaire

Subject Satisfaction Questionnaire					
Subject ID:					
Date: ____ / ____ / 20____ (MM / DD / YY)					
Please answer the following questions (tick one box per row).					
Statement	Strongly agree	Agree	Neither agree nor disagree	Disagree	Strongly disagree
1. I have less TMJ pain (pain in my jaw) after the treatments.	☐	☐	☐	☐	☐
2. My jaw feels more relaxed after the treatments.	☐	☐	☐	☐	☐
3. The stiffness in my jaw has significantly reduced after the treatments.	☐	☐	☐	☐	☐
4. I feel less frustrated with TMJ pain after the treatments.	☐	☐	☐	☐	☐
5. I have less pain in my neck after the treatments.	☐	☐	☐	☐	☐
6. I have less pain while eating and/or chewing after the treatments.	☐	☐	☐	☐	☐
7. I have less pain while opening my mouth/yawning after the treatments.	☐	☐	☐	☐	☐
8. I have fewer headaches after the treatments.	☐	☐	☐	☐	☐
9. Moving my mouth is easier after the treatments.	☐	☐	☐	☐	☐
10. My jaw feels less tender after the treatments.	☐	☐	☐	☐	☐
11. The clicking in my jaw has reduced after the treatments.	☐	☐	☐	☐	☐
12. I am satisfied with the treatment outcomes.	☐	☐	☐	☐	☐
